# Dose–response relationships of sarcopenia parameters with incident disability and mortality in older Japanese adults

**DOI:** 10.1002/jcsm.12958

**Published:** 2022-02-25

**Authors:** Satoshi Seino, Akihiko Kitamura, Takumi Abe, Yu Taniguchi, Hiroshi Murayama, Hidenori Amano, Mariko Nishi, Yu Nofuji, Yuri Yokoyama, Miki Narita, Shoji Shinkai, Yoshinori Fujiwara

**Affiliations:** ^1^ Research Team for Social Participation and Community Health Tokyo Metropolitan Institute of Gerontology Tokyo Japan; ^2^ Health Town Development Science Center Yao City Health Center Osaka Japan; ^3^ Integrated Research Initiative for Living Well with Dementia Tokyo Metropolitan Institute of Gerontology Tokyo Japan; ^4^ Center for Health and Environmental Risk Research National Institute for Environmental Studies Ibaraki Japan; ^5^ Department of Nutrition Sciences Kagawa Nutrition University Saitama Japan

**Keywords:** Sarcopenia, Skeletal muscle, Handgrip strength, Gait speed, Disability, Mortality

## Abstract

**Background:**

Sarcopenia‐related parameters may have differential impacts on health‐related outcomes in older adults. We examined dose–response relationships of body composition, muscle strength, and physical performance with incident disability and mortality.

**Methods:**

This prospective study included 1765 Japanese residents (862 men; 903 women) aged ≥65 years who participated in health check‐ups. Outcomes were incident disability and all‐cause mortality. Fat mass index (FMI) and skeletal muscle mass index (SMI), determined using segmental multi‐frequency bioelectrical impedance analysis, handgrip strength (HGS), and usual gait speed (UGS) were measured. We determined multivariate‐adjusted hazard ratios (HRs) for disability and mortality relative to sex‐specific reference values (FMI: medians; SMI: 7.0 kg/m^2^ for men and 5.7 kg/m^2^ for women; HGS: 28 kg for men and 18 kg for women; or UGS: 1.0 m/s for both sexes). Association shapes were examined using restricted cubic splines or fractional polynomial functions.

**Results:**

The median follow‐up was 5.3 years; 107 (12.7%) men and 123 (14.2%) women developed disability, and 101 (11.7%) men and 56 (6.2%) women died. FMI did not impact any outcome in men and disability in women, while an FMI ≤ 7.3 kg/m^2^ (median) was significantly associated with higher mortality risk in women, compared with median FMI. SMI did not impact disability in either sex and mortality in women, but showed a significant inverse dose–response relationship with mortality risk in men [HRs (95% confidence intervals) of minimum and maximum values compared with the reference value: 2.18 (1.07–4.46) and 0.43 (0.20–0.93), respectively], independent of HGS and UGS. HGS and UGS showed a significant inverse dose–response relationship with disability in both sexes [HGS: 1.71 (1.00–2.91) and 0.31 (0.09–0.99), respectively, in men, 2.42 (1.18–4.96) and 0.41 (0.20–0.85), respectively, in women; UGS: 2.14 (1.23–3.74) and 0.23 (0.08–0.67), respectively, in men, 3.26 (2.07–5.14) and 0.11 (0.05–0.26), respectively, in women] and mortality in women [HGS: 6.84 (2.84–16.47) and 0.06 (0.02–0.21), respectively; UGS: 2.67 (1.14–6.27) and 0.30 (0.11–0.85), respectively], independent of body composition, but did not impact mortality in men.

**Conclusions:**

Disability risk was more dependent on muscle strength and physical performance in both sexes. Mortality risk in men was more dependent on muscle mass, and mortality risk in women was influenced by lower fat mass along with muscle strength and physical performance. Although improving muscle strength and physical performance should be the first target for health promotion, it is also necessary to pay attention to body composition to extend life expectancy in older adults.

## Introduction

Sarcopenia was originally defined as an age‐related loss of skeletal muscle mass in older adults.^1^, ^2^ Since 2010, several working groups have proposed conceptual and operational definitions of sarcopenia, including muscle mass, muscle strength, and/or physical performance.^3^, ^4^, ^5^, ^6^, ^7^, ^8^, ^9^ In 2020, the Sarcopenia Definition and Outcomes Consortium^10^ stated that weakness, defined by low handgrip strength (HGS), as well as slowness, defined by low usual gait speed (UGS), should be included in the definition of sarcopenia. However, they were sceptical about including the muscle mass. Thus, the focus of the definition of sarcopenia has shifted from skeletal muscle mass to strength and physical performance over the last two decades.

These trends are attributable to the evidence showing that muscle strength and physical performance are more strongly associated with health‐related outcomes than muscle mass.^11^, ^12^ Although previous studies have shown that muscle mass only partially accounts for the muscle strength–mortality association,^13^ evidence on whether muscle mass–outcome associations are mediated by muscle strength and physical performance is limited.^14^ Moreover, fat mass is a critical confounder of these associations.^14^, ^15^ However, many of the studies^11^, ^12^ have not sufficiently accounted for this impact. Additionally, there is an absolute shortage of Asian data in previous findings.^11^, ^12^ Population‐specific investigations are warranted because the characteristics of body composition and prevalence of obesity vary by population.^16^ Finally, although the associations of body composition, muscle strength, and physical performance with health‐related outcomes have been examined using categorical or linear approaches in previous studies,^13^, ^14^, ^17^, ^18^ the true shape of the association is unknown. If the true shape is not linear, these approaches may mask or weaken significant associations. The cubic spline analysis, which has high precision,^19^ reveals a more elaborate shape of the association than that possible with these approaches.

Therefore, we examined the shapes of the associations of body composition, muscle strength, and physical performance, after accounting for the confounding effects of each on the other, with incident disability and all‐cause mortality among community‐dwelling older Japanese adults. Specifically, we aimed to clarify (i) whether body composition, muscle strength, and physical performance have differential impacts on incident disability and mortality and (ii) whether their impacts differ between sexes because of the previously established sex‐related differences in body composition, muscle strength, and physical performance.^20^

## Methods

### Study population

We used combined data^21^, ^22^ from the Kusatsu Longitudinal Study^23^ and the Hatoyama Cohort Study.^24^ We extracted the baseline data of 1944 participants (1250 from Kusatsu and 694 from Hatoyama) aged ≥ 65 years, for whom information on body composition, muscle strength, and physical performance was collected at an initial check‐up between 2008 and 2016 in Kusatsu and 2010 and 2014 in Hatoyama. A total of 179 participants were excluded because of non‐standard body composition findings or missing data. Ultimately, 1765 participants (862 men and 903 women) without disabilities were included.

### Measurements

#### Body composition parameters

Body composition parameters were measured using direct segmental multi‐frequency bioelectrical impedance analysis (InBody 720 analyser, InBody Co. Ltd., Seoul, Korea)^25^ using a tetrapolar, 8‐point tactile electrode system that separately measures impedance of the arms, trunk, and legs at six different frequencies (1, 5, 50, 250, 500, and 1000 kHz). The InBody 720 automatically estimates weight, body mass index (BMI), fat mass, and lean soft‐tissue mass (LSTM) of the arms and legs. The appendicular LSTM was calculated as the sum of the LSTM of the arms and legs. To determine the fat mass index (FMI)^26^ and skeletal muscle mass index (SMI),^2^ the fat mass and appendicular LSTM were normalized by height in meters squared.

### Muscle strength and physical performance

To measure muscle strength, HGS was assessed using a Smedley‐type hand dynamometer (Yagami Co., Tokyo, Japan).^27^ Participants stood with their arms hanging naturally at their sides, holding the dynamometer with a grip size adjusted to a comfortable level. They were instructed and verbally encouraged to squeeze the dynamometer as hard as possible.^27^ Participants performed two trials with the dominant hand, and the best result (to the nearest 0.1 kg) was used.

For physical performance measurements, UGS was measured over a distance of 5 m, with acceleration and deceleration phases of 3 m each.^27^ Participants were instructed to stand with their feet behind but just touch a stationary starting line marked with a tape strip at 0 m. On the tester's command, they were to start walking at their normal pace along an 11‐m course. The actual walking time was measured over 5 m and was started when the participant's trunk had passed the 3‐m mark and ended when the trunk was beyond the 8‐m mark.^27^ UGS was measured only once and calculated as the distance divided by the time taken to walk that distance (m/s).

### All‐cause mortality and disability

We ascertained the occurrence of disability and/or all deaths on 13 December 2017, in Kusatsu town and 31 December 2015, in Hatoyama town. All‐cause mortality was confirmed by checking local registries that have linked records with the Japanese National Vital Statistics System.

Disability was identified in the participants using the nationally unified database of the long‐term care insurance (LTCI) system, enrolment in which is mandatory for all Japanese adults aged ≥ 40 years. The system provides formal care and support for eligible Japanese adults aged ≥ 65 years with physical and mental disabilities.^28^, ^29^ LTCI certification is based on a nationally standardized multistep assessment.^28^, ^29^ Ultimately, the Municipal Certification Committee of Needed Long‐Term Care decides whether an older adult should be certified as requiring long‐term care and classifies care needs under one of seven levels (support level, 1–2; care level, 1–5). Disability was defined as the onset of long‐term care needs at the support level 1 or above,^30^ using the date of the LTCI application as the date of the disability incident.^22^

### Covariates

The covariates included baseline age, study area (Kusatsu or Hatoyama), year of first visit for health check‐up, alcohol consumption and tobacco smoking status (current, never, or former), previous diagnosis of stroke, heart disease, cancer, hypertension (systolic blood pressure ≥ 140 mmHg and/or diastolic blood pressure ≥ 90 mmHg; previous diagnosis or ongoing medical treatment), and diabetes (haemoglobin A1c ≥ 6.5%; previous diagnosis or ongoing medical treatment), high total cholesterol (≥240 mg/dL or ongoing medical treatment), low total cholesterol (<160 mg/dL), hypoalbuminemia (<3.8 g/dL), anaemia (haemoglobin < 13.0 g/dL in men or <12.0 g/dL in women), and chronic kidney disease (estimated glomerular filtration rate < 60 mL/min/1.73 m^2^, calculated using creatinine concentration and equations developed for Japanese adults^31^), low activity (an answer of ‘less than once a day’ to the question ‘How often do you usually go outdoors?’^22^), depressed mood (short form of the Geriatric Depression Scale ≥ 5^32^), and cognitive impairment (Mini‐Mental State Examination score ≤ 23^33^).

### Statistical analyses

All data were analysed by sex using Stata 16.1 (StataCorp, TX, USA). Statistical significance was set at *α* = 0.05. Descriptive statistics were used to characterize the participants. The unpaired *t‐*test, Mann–Whitney *U*‐test, and *χ* ^2^ test were used to compare the baseline characteristics of independent participants and those with disability or death.

For primary analysis, we used the Cox proportional hazards model, with incident disability or mortality as the dependent variable and FMI, SMI, HGS, and UGS as independent variables. We then constructed two multivariate analytic models and examined multivariate‐adjusted hazard ratios (HRs) and 95% confidence intervals (CIs) in terms of FMI, SMI, HGS, and UGS for incident disability or mortality. Model 1 was adjusted for baseline age, study area, year of first visit for health check‐up, alcohol consumption and smoking status, hypertension, stroke, heart disease, diabetes, cancer, high total cholesterol, low total cholesterol, hypoalbuminemia, anaemia, chronic kidney disease, low activity, depressed mood, and cognitive impairment. In the analyses with FMI and SMI as independent variables, these two indices were mutually adjusted as were other covariates because they are mutual confounders and both have sex‐specific differences.^15^, ^34^ Moreover, to examine the independent impacts of body composition, muscle strength, and physical performance on outcomes, Model 2 was adjusted for HGS and UGS in the analysis with FMI or SMI as an independent variable or with FMI and SMI in the analysis with HGS or UGS as an independent variable, in addition to the variables in Model 1.

Furthermore, using fractional polynomial (FP) functions or restricted cubic spline (RCS), in accordance with the procedures of previous studies,^21^, ^35^ we examined the dose–response relationship of FMI, SMI, HGS, and UGS independently with incident disability or mortality risk. For the FP procedure, the power transformation was performed by setting the default values of Stata, namely, −2, −1, −0.5, 0, 0.5, 1, 2, and 3. For the RCS, the knot locations were chosen as 3, 4, or 5. We used the Akaike information criterion to select the FP transformation or RCS with 3, 4, or 5 knots and adopted the model with the lowest Akaike information criterion value.^36^ We set the median FMI or cut‐off points for sarcopenia criteria defined by the Asian Working Group for Sarcopenia in 2019 (SMI: 7.0 kg/m^2^ for men and 5.7 kg/m^2^ for women; HGS: 28 kg for men and 18 kg for women; or UGS: 1.0 m/s for both sexes^9^), as the reference value for each model.

In the secondary analyses of the possible influence of reverse causation on the association between each measure and disability or mortality, we performed sensitivity analyses using the same statistical approach, after excluding disability or death that occurred during the first year and the first 2 years of follow‐up.

## Results

During a median (interquartile range) follow‐up of 5.3 (3.5–6.9) years, 107 (12.7%) men and 123 (14.2%) women presented with functional disabilities, with disability rates of 24.3 and 25.5 per 1000 person‐years, respectively. Of these, 9 (8.4%) men and 14 (11.4%) women presented with disabilities during the first year, and 22 (20.6%) men and 26 (21.1%) women presented with disabilities during the first 2 years. During a median follow‐up of 5.3 (4.4–7.5) years, 101 (11.7%) men and 56 (6.2%) women died, with mortality rates of 21.5 and 10.4 per 1000 person‐years, respectively. Of these, 2 (2.0%) men and 2 (3.6%) women died during the first year, and 6 (5.9%) men and 5 (8.9%) women died during the first 2 years.

*Tables* 1, 2 show the baseline characteristics of the study population according to sex and disability status (*Table* 1) and according to sex and survival status (*Table* 2). Independent participants and those with disability or death consistently and significantly differed in baseline age, study area, total cholesterol and albumin levels, estimated glomerular filtration rate, Mini‐Mental State Examination score, height, weight, appendicular LSTM, SMI, HGS, UGS, and prevalence of low total cholesterol in both sexes. In men, independent participants and those with disability significantly differed in alcohol consumption status, haemoglobin A1c, and prevalence of diabetes and high total cholesterol (*Table* 1), while survivors and non‐survivors showed significant differences in haemoglobin and BMI (*Table* 2). Consistent and significant differences were observed in diastolic blood pressure and the prevalence of hypoalbuminemia, anaemia and chronic kidney disease in men (*Tables* 1, 2). In women, independent participants and those with disability significantly differed in haemoglobin level and the prevalence of hypertension, stroke, and anaemia (*Table* 1), while survivors and non‐survivors significantly differed in diastolic blood pressure and the prevalence of hypoalbuminemia and chronic kidney disease (*Table* 2). Consistent and significant differences were observed in the Geriatric Depression Scale scores and the prevalence of high total cholesterol, low activity, depressive symptoms, and cognitive impairment (*Tables* 1, 2).

**Table 1:** Baseline characteristics of the study population according to sex and disability status

Variables	Mean ± SD or *n* (%)					
	Men (*n* = 844)			Women (*n* = 867)		
	No incident disability	Incident disability	*P* value	No incident disability	Incident disability	*P* value
	(*n* = 737)	(*n* = 107)		(*n* = 744)	(*n* = 123)	
Age (years)	70.8 ± 4.8	78.0 ± 6.2	**<0.001**	70.5 ± 5.2	76.9 ± 6.1	**<0.001**
65–74	574 (77.9)	28 (26.2)	**<0.001**	579 (77.8)	41 (33.3)	**<0.001**
75^+^	163 (22.1)	79 (73.8)		165 (22.2)	82 (66.7)	
Study area			**0.001**			**0.001**
Kusatsu	414 (56.2)	83 (77.6)		510 (68.6)	102 (82.9)	
Hatoyama	323 (43.8)	24 (22.4)		234 (31.4)	21 (17.1)	
Alcohol consumption status			**0.020**			0.09
Current	514 (69.7)	61 (57.0)		295 (39.7)	36 (29.3)	
Former	71 (9.6)	10 (9.4)		53 (7.1)	8 (6.5)	
Never	117 (15.9)	26 (24.3)		356 (47.9)	68 (55.3)	
Missing	35 (4.8)	10 (9.4)		40 (5.4)	11 (8.9)	
Smoking status			0.16			0.39
Current	146 (19.8)	22 (20.6)		64 (8.6)	12 (9.8)	
Former	361 (49.0)	44 (41.1)		66 (8.9)	8 (6.5)	
Never	195 (26.4)	31 (29.0)		573 (77.0)	92 (74.8)	
Missing	35 (4.8)	10 (9.3)		41 (5.5)	11 (8.9)	
SBP (mmHg)	138 ± 22	138 ± 20	0.69	136 ± 22	138 ± 23	0.18
DBP (mmHg)	80 ± 12	78 ± 12	**0.026**	77 ± 12	76 ± 12	0.26
Hypertension^a^	490 (66.5)	72 (67.3)	0.87	442 (59.4)	92 (74.8)	**0.001**
Stroke	41 (5.6)	11 (10.3)	0.06	25 (3.4)	9 (7.3)	**0.036**
Heart disease	120 (16.3)	19 (17.8)	0.70	80 (10.8)	10 (8.1)	0.38
Cancer	75 (10.2)	12 (11.2)	0.74	59 (7.9)	8 (6.5)	0.58
Haemoglobin A1c (%)	5.5 ± 0.7	5.8 ± 1.1	**<0.001**	5.5 ± 0.6	5.4 ± 0.6	0.27
Diabetes^b^	143 (19.4)	34 (31.8)	**0.003**	80 (10.8)	18 (14.6)	0.21
Total cholesterol (mg/dL)	198 ± 33	190 ± 38	**0.021**	215 ± 34	203 ± 38	**<0.001**
High total cholesterol^c^	180 (24.4)	17 (15.9)	**0.049**	346 (46.5)	44 (35.8)	**0.027**
Low total cholesterol^d^	87 (11.8)	21 (19.6)	**0.024**	29 (3.9)	10 (8.1)	**0.036**
Albumin (g/dL)	4.3 ± 0.2	4.1 ± 0.3	**<0.001**	4.3 ± 0.3	4.2 ± 0.3	**<0.001**
Hypoalbuminemia^e^	34 (4.6)	22 (20.6)	**<0.001**	32 (4.3)	8 (6.5)	0.28
Haemoglobin (g/dL)	14.4 ± 1.3	14.1 ± 1.6	0.06	13.2 ± 1.1	12.9 ± 1.3	**0.007**
Anaemia^f^	99 (13.4)	22 (20.6)	**0.049**	80 (10.8)	21 (17.1)	**0.043**
eGFR (mL/min/1.73 m^2^)	68.8 ± 13.9	62.8 ± 14.0	**<0.001**	68.2 ± 13.4	65.0 ± 17.9	**0.024**
Chronic kidney disease^g^	185 (25.1)	43 (40.2)	**0.001**	193 (25.9)	41 (33.3)	0.09
Low activity^h^	20 (2.7)	4 (3.7)	0.55	27 (3.6)	11 (8.9)	**0.008**
GDS score	2.7 ± 2.6	3.1 ± 2.5	0.11	2.9 ± 2.7	4.3 ± 3.1	**<0.001**
Depressive symptoms^i^	151 (20.5)	27 (25.2)	0.26	171 (23.0)	45 (36.6)	**0.001**
MMSE score	27.9 ± 2.3	26.7 ± 2.7	**<0.001**	28.3 ± 2.1	26.6 ± 3.9	**<0.001**
Cognitive impairment^j^	31 (4.2)	9 (8.4)	0.06	20 (2.7)	15 (12.2)	**<0.001**
Height (cm)	163.0 ± 5.8	160.2 ± 6.6	**<0.001**	150.3 ± 5.5	146.8 ± 6.1	**<0.001**
Weight (kg)	62.5 ± 9.0	59.6 ± 8.9	**0.002**	51.9 ± 7.9	49.7 ± 8.2	**0.004**
BMI (kg/m^2^)	23.5 ± 2.9	23.2 ± 2.8	0.27	23.0 ± 3.2	23.0 ± 3.4	0.93
Fat mass (kg)	15.4 ± 5.5	15.8 ± 5.6	0.43	16.8 ± 5.8	16.4 ± 5.9	0.40
FMI (kg/m^2^)	5.8 ± 2.1	6.2 ± 2.2	0.07	7.5 ± 2.5	7.6 ± 2.8	0.57
Appendicular lean soft‐tissue mass (kg)	19.7 ± 2.7	18.0 ± 2.9	**<0.001**	13.5 ± 2.0	12.4 ± 2.3	**<0.001**
SMI (kg/m^2^)	7.4 ± 0.7	7.0 ± 0.7	**<0.001**	6.0 ± 0.6	5.8 ± 0.8	**0.003**
HGS (kg)	35.2 ± 6.3	30.0 ± 6.3	**<0.001**	21.8 ± 4.3	18.1 ± 5.1	**<0.001**
UGS (m/s)	1.36 ± 0.23	1.24 ± 0.23	**<0.001**	1.36 ± 0.23	1.16 ± 0.25	**<0.001**

BMI, body mass index; DBP, diastolic blood pressure; eGFR, estimated glomerular filtration rate; FMI, fat mass index; GDS, Geriatric Depression Scale; HGS, handgrip strength; MMSE, Mini‐Mental State Examination; SBP, systolic blood pressure; SD, standard deviation; SMI, skeletal muscle mass index; UGS, usual gait speed.

^a^ Hypertension: SBP ≥ 140 mmHg and/or DBP ≥ 90 mmHg or previous diagnosis or current medical treatment.

^b^ Diabetes: haemoglobin A1c ≥ 6.5% or current medical treatment.

^c^ High total cholesterol: ≥240 mg/dL or current medical treatment.

^d^ Low total cholesterol: <160 mg/dL.

^e^ Hypoalbuminemia: albumin <3.8 g/dL.

^f^ Anaemia: haemoglobin < 13.0 g/dL in men and <12.0 g/dL in women.

^g^ Chronic kidney disease: eGFR < 60 mL/min/1.73 m^2^.

^h^ Low activity: going outdoors < 1 time/week.

^i^ Depressive mood (GDS score ≥ 5).

^j^ Cognitive impairment (MMSE score ≤ 23).

**Table 2:** Baseline characteristics of the study population according to sex and survival status

Variables	Mean ± SD or *n* (%)					
	Men (*n* = 862)			Women (*n* = 903)		
	Survivors	Non‐survivors	*P* value	Survivors	Non‐survivors	*P* value
	(*n* = 761)	(*n* = 101)		(*n* = 847)	(*n* = 56)	
Age (years)	71.2 ± 5.4	76.6 ± 6.5	**<0.001**	71.4 ± 5.7	77.6 ± 8.3	**<0.001**
65–74	572 (75.2)	36 (35.6)	**<0.001**	607 (71.7)	20 (35.7)	**<0.001**
75^+^	189 (24.8)	65 (64.4)		240 (28.3)	36 (64.3)	
Study area			**<0.001**			**0.014**
Kusatsu	426 (56.0)	87 (86.1)		596 (70.4)	48 (85.7)	
Hatoyama	335 (44.0)	14 (13.9)		251 (29.6)	8 (14.3)	
Alcohol consumption status			0.17			0.13
Current	522 (68.6)	61 (60.4)		325 (38.4)	16 (28.6)	
Former	71 (9.3)	15 (14.9)		62 (7.3)	2 (3.6)	
Never	130 (17.1)	17 (16.8)		409 (48.3)	36 (64.3)	
Missing	38 (5.0)	8 (7.9)		51 (6.0)	2 (3.6)	
Smoking status			0.49			0.39
Current	149 (19.6)	23 (22.8)		73 (8.6)	5 (8.9)	
Former	372 (48.9)	44 (43.6)		69 (8.2)	8 (14.3)	
Never	202 (26.5)	26 (25.7)		653 (77.1)	41 (73.2)	
Missing	38 (5.0)	8 (7.9)		52 (6.1)	2 (3.6)	
SBP (mmHg)	138 ± 22	135 ± 21	0.26	136 ± 22	137 ± 23	0.54
DBP (mmHg)	80 ± 12	76 ± 13	**<0.001**	77 ± 12	73 ± 14	**0.020**
Hypertension^a^	509 (66.9)	66 (65.4)	0.76	523 (61.8)	35 (62.5)	0.91
Stroke	46 (6.0)	8 (7.9)	0.47	37 (4.4)	3 (5.4)	0.73
Heart disease	121 (15.9)	21 (20.8)	0.21	84 (9.9)	10 (17.9)	0.06
Cancer	78 (10.3)	12 (11.9)	0.61	65 (7.7)	6 (10.7)	0.41
Haemoglobin A1c (%)	5.5 ± 0.8	5.6 ± 0.8	0.38	5.5 ± 0.5	5.4 ± 1.2	0.89
Diabetes^b^	152 (20.0)	27 (26.7)	0.12	96 (11.3)	6 (10.7)	0.89
Total cholesterol (mg/dL)	198 ± 34	184 ± 35	**<0.001**	214 ± 35	198 ± 41	**<0.001**
High total cholesterol^c^	180 (23.7)	20 (19.8)	0.39	388 (45.8)	17 (30.3)	**0.024**
Low total cholesterol^d^	84 (11.0)	29 (28.7)	**<0.001**	36 (4.3)	6 (10.7)	**0.026**
Albumin (g/dL)	4.3 ± 0.3	4.1 ± 0.3	**<0.001**	4.3 ± 0.2	4.1 ± 0.4	**<0.001**
Hypoalbuminemia^e^	41 (5.4)	18 (17.8)	**<0.001**	33 (3.9)	12 (21.4)	**<0.001**
Haemoglobin (g/dL)	14.4 ± 1.3	14.0 ± 1.5	**0.021**	13.2 ± 1.1	12.9 ± 1.2	0.14
Anaemia^f^	103 (13.5)	22 (21.8)	**0.027**	100 (11.8)	10 (17.9)	0.18
eGFR (mL/min/1.73 m^2^)	68.5 ± 14.0	63.6 ± 14.7	**0.001**	67.8 ± 14.0	62.4 ± 16.3	**0.007**
Chronic kidney disease^g^	195 (25.6)	41 (40.6)	**0.002**	226 (26.7)	28 (50.0)	**<0.001**
Low activity ^h^	21 (2.8)	4 (4.0)	0.50	35 (4.1)	7 (12.5)	**0.004**
GDS score	2.7 ± 2.7	3.2 ± 2.7	0.08	3.1 ± 2.8	4.1 ± 3.1	**0.009**
Depressive symptoms^i^	158 (20.8)	29 (28.7)	0.07	214 (25.3)	21 (37.5)	**0.043**
MMSE score	27.8 ± 2.4	26.8 ± 2.9	**<0.001**	28.1 ± 2.6	26.6 ± 4.2	**<0.001**
Cognitive impairment^j^	36 (4.7)	9 (8.9)	0.08	36 (4.3)	7 (12.5)	**0.005**
Height (cm)	162.9 ± 5.9	159.7 ± 6.3	**<0.001**	149.7 ± 5.8	146.9 ± 6.6	**<0.001**
Weight (kg)	62.6 ± 9.0	57.6 ± 8.6	**<0.001**	51.6 ± 7.9	48.6 ± 8.5	**0.006**
BMI (kg/m^2^)	23.6 ± 2.9	22.6 ± 3.0	**0.001**	23.0 ± 3.2	22.5 ± 3.5	0.23
Fat mass (kg)	15.5 ± 5.5	14.9 ± 5.6	0.26	16.8 ± 5.8	15.7 ± 5.9	0.18
FMI (kg/m^2^)	5.9 ± 2.1	5.9 ± 2.2	0.95	7.5 ± 2.6	7.3 ± 2.7	0.53
Appendicular lean soft‐tissue mass (kg)	19.7 ± 2.7	17.5 ± 2.6	**<0.001**	13.3 ± 2.1	12.3 ± 2.5	**<0.001**
SMI (kg/m^2^)	7.4 ± 0.7	6.9 ± 0.7	**<0.001**	5.9 ± 0.7	5.7 ± 0.9	**0.004**
HGS (kg)	35.0 ± 6.5	29.8 ± 6.6	**<0.001**	21.3 ± 4.5	16.0 ± 5.4	**<0.001**
UGS (m/s)	1.35 ± 0.23	1.26 ± 0.23	**<0.001**	1.33 ± 0.25	1.10 ± 0.30	**<0.001**

BMI, body mass index; DBP, diastolic blood pressure; eGFR, estimated glomerular filtration rate; FMI, fat mass index; GDS, Geriatric Depression Scale; HGS, handgrip strength; MMSE, Mini‐Mental State Examination; SBP, systolic blood pressure; SD, standard deviation; SMI, skeletal muscle mass index; UGS, usual gait speed.

^a^ Hypertension: SBP ≥ 140 mmHg and/or DBP ≥ 90 mmHg or previous diagnosis or current medical treatment.

^b^ Diabetes: haemoglobin A1c ≥ 6.5% or current medical treatment.

^c^ High total cholesterol: ≥240 mg/dL or current medical treatment.

^d^ Low total cholesterol: <160 mg/dL.

^e^ Hypoalbuminemia: albumin < 3.8 g/dL.

^f^ Anaemia: haemoglobin < 13.0 g/dL in men and <12.0 g/dL in women.

^g^ Chronic kidney disease: eGFR < 60 mL/min/1.73 m^2^.

^h^ Low activity: going outdoors < 1 time/week.

^i^ Depressive mood (GDS score ≥ 5).

^j^ Cognitive impairment (MMSE score ≤ 23).

**Figure 1:**
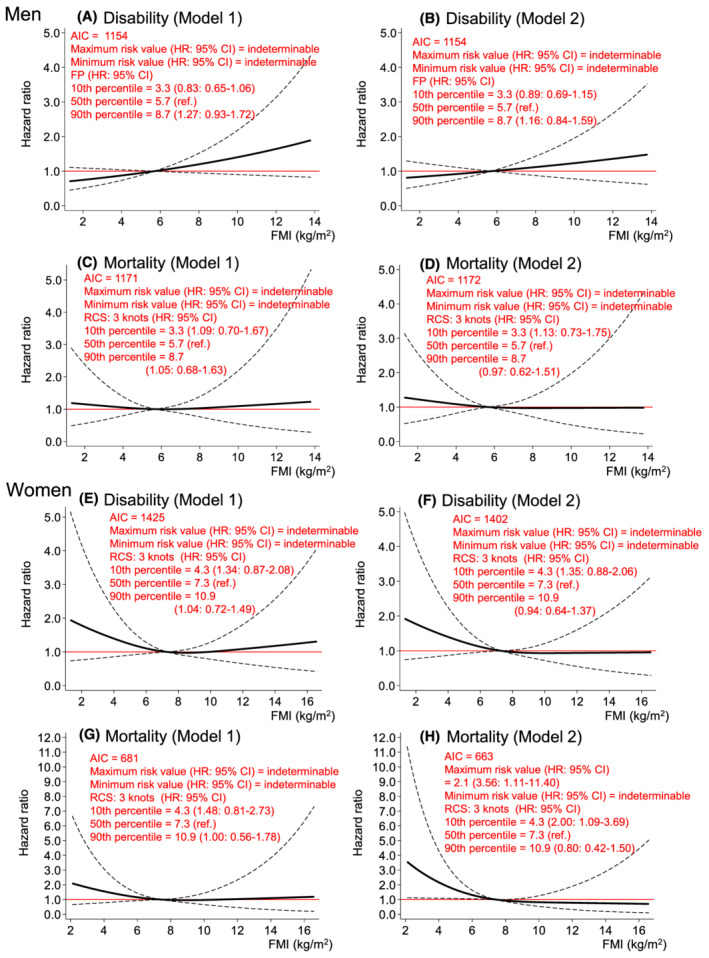
Dose–response relationships of FMI with incident disability and mortality risk. *Figure* 1A–1D shows the relationships of FMI with disability (*Figure* 1A–1B) and mortality (*Figure* 1C–1D) risks in men. *Figure* 1E–1H shows the relationships of FMI with disability (*Figure* 1E–1F) and mortality (*Figure* 1G–1H) risks in women. *Figure* 1A–1B was modelled using an FP function, and *Figure* 1C–1H was modelled using an RCS with three knots located at the 10th, 50th, and 90th percentiles of the distribution of the index. Model 1 was adjusted for baseline age, study area, year of first visit for health check‐up, alcohol consumption and smoking status, hypertension, stroke, heart disease, diabetes, cancer, high total cholesterol, low total cholesterol, hypoalbuminemia, anaemia, chronic kidney disease, low activity, depressed mood, cognitive impairment, and SMI. Model 2 was adjusted for the variables in Model 1, plus HGS and UGS. The reference value for each model is the median FMI (i.e. FMI of 5.7 kg/m^2^ in men and FMI of 7.3 kg/m^2^ in women). The dashed lines indicate the 95% confidence intervals. AIC, Akaike's information criterion; FMI, fat mass index; FP, fractional polynomial; HGS, handgrip strength; HR, hazard ratio; SMI, skeletal muscle mass index; UGS, usual gait speed.

*Figure* 1 shows the dose–response relationships of FMI with incident disability and mortality risks. FMI consistently had no impact on disability and mortality in the first multivariate model (Model 1) and the additional adjustment model for HGS and UGS (Model 2) in men (*Figure* 1A–1D). In women, although FMI had no impact on disability in both models (*Figure* 1E–1F) and mortality in Model 1 (*Figure* 1G), FMI ≤ 7.3 kg/m^2^ (median) was significantly associated with higher mortality risk in Model 2 [HR (95% CI) of minimum value (2.1 kg/m^2^) compared with reference value (7.3 kg/m^2^): 3.56 (1.11–11.40) (*Figure* 1H)].

**Figure 2:**
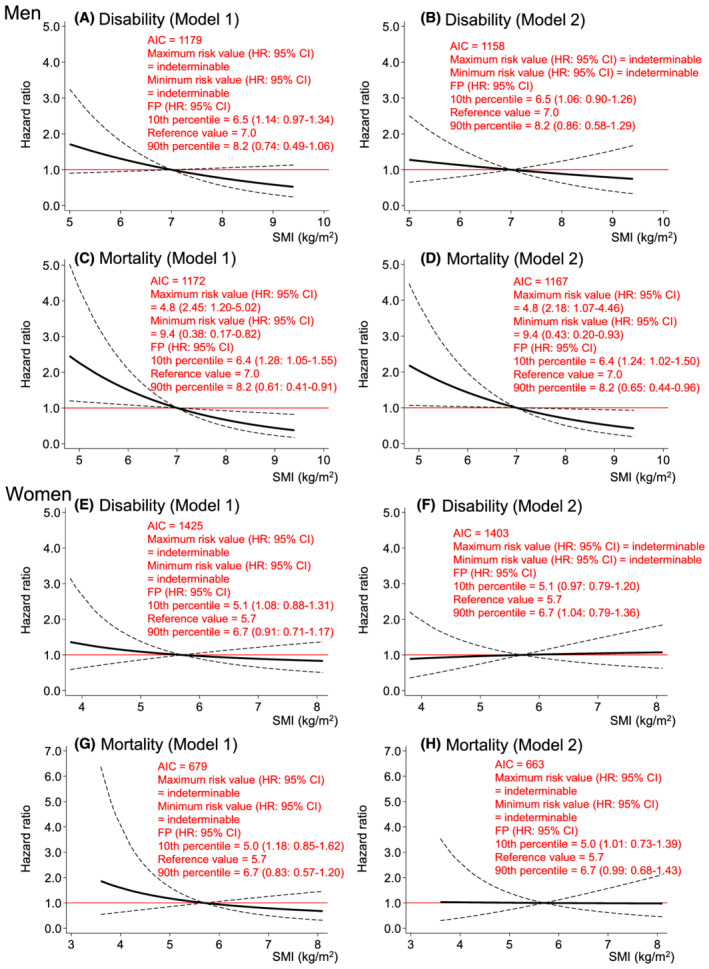
Dose–response relationships of SMI with incident disability and mortality risk. *Figure* 2A–2D shows the relationships of SMI with disability (*Figure* 2A–2B) and mortality (*Figure* 2C–2D) risks in men. *Figure* 2E–2H shows the relationships of SMI with disability (*Figure* 2E–2F) and mortality (*Figure* 2G–2H) risks in women. *Figure* 2A–2H was modelled using an FP function. Model 1 was adjusted for baseline age, study area, year of first visit for health check‐up, alcohol consumption and smoking status, hypertension, stroke, heart disease, diabetes, cancer, high total cholesterol, low total cholesterol, hypoalbuminemia, anaemia, chronic kidney disease, low activity, depressed mood, cognitive impairment, and FMI. Model 2 was adjusted for the variables in Model 1, plus HGS and UGS. The reference values for each model are the cut‐off points for sarcopenia criteria defined by the Asian Working Group for Sarcopenia in 2019 (i.e. SMI of 7.0 kg/m^2^ in men and SMI of 5.7 kg/m^2^ in women). The dashed lines indicate the 95% confidence intervals. AIC, Akaike's information criterion; FMI, fat mass index; FP, fractional polynomial; HGS, handgrip strength; HR, hazard ratio; SMI, skeletal muscle mass index; UGS, usual gait speed.

*Figure* 2 shows the dose–response relationships of SMI with incident disability and mortality risks. SMI did not impact disability in either sex and mortality in women in both models (*Figure* 2A, 2B, and 2E–2H). However, SMI had significant inverse dose–response relationships with mortality risk consistently in both Models in men [HRs (95% CIs) of minimum (4.8 kg/m^2^) and maximum (9.4 kg/m^2^) values compared with reference value (7.0 kg/m^2^): 2.45 (1.20–5.02) and 0.38 (0.17–0.82), respectively in model 1 (*Figure* 2C); 2.18 (1.07–4.48) and 0.43 (0.20–0.93), respectively in model 2(*Figure* 2D)].

**Figure 3:**
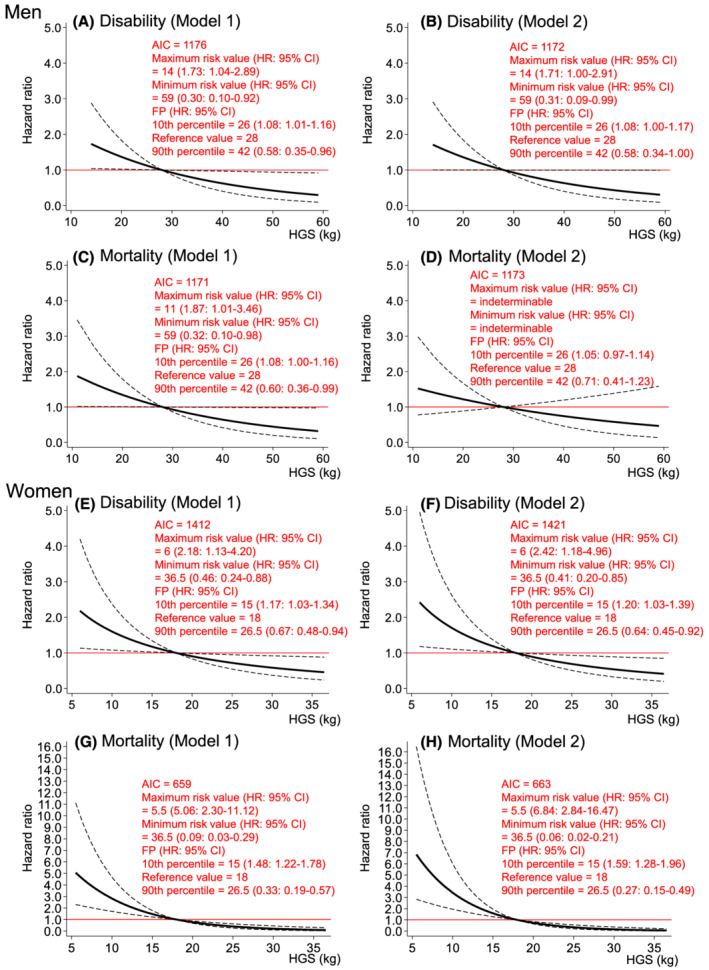
Dose–response relationships of HGS with incident disability and mortality risk. *Figure* 3A–3D shows the relationships of HGS with disability (*Figure* 3A–3B) and mortality (*Figure* 3C–3D) risks in men. *Figure* 3E–3H shows the relationships of HGS with disability (*Figure* 3E–3F) and mortality (*Figure* 3G–3H) risks in women. *Figure* 3A–3H was modelled using an FP function. Model 1 was adjusted for baseline age, study area, year of first visit for health check‐up, drinking and smoking status, hypertension, stroke, heart disease, diabetes, cancer, high total cholesterol, low total cholesterol, hypoalbuminemia, anaemia, chronic kidney disease, low activity, depressed mood, and cognitive impairment. Model 2 was adjusted for the variables in Model 1, plus FMI and SMI. The reference values for each model are the cut‐off points for sarcopenia criteria defined by the Asian Working Group for Sarcopenia in 2019 (i.e. HGS of 28 kg in men and HGS of 18 kg in women). The dashed lines indicate the 95% confidence intervals. AIC, Akaike's information criterion; FMI, fat mass index; FP, fractional polynomial; HGS, handgrip strength; HR, hazard ratio; SMI, skeletal muscle mass index.

*Figure* 3 shows the dose–response relationships of HGS with incident disability and mortality risks. HGS had significant inverse dose–response relationships with disability and mortality risk consistently in Model 1 in both sexes (*Figure* 3A, 3C, 3E, and 3G). Although the significant inverse HGS–disability association persisted even after further adjustment for FMI and SMI in men [HRs (95% CIs) of minimum (14 kg) and maximum (59 kg) values compared with reference value (28 kg): 1.71 (1.00–2.91) and 0.31 (0.09–0.99), respectively (*Figure* 3B)], the significant HGS–mortality association disappeared (*Figure* 3D). HGS in women consistently showed significant inverse dose–response relationships with disability [HRs (95% CIs) of minimum (6 kg) and maximum (36.5 kg) values compared with the reference value (18 kg): 2.42 (1.18–4.96) and 0.41 (0.20–0.85), respectively] and mortality [HRs (95% CIs) of minimum (5.5 kg) and maximum (36.5 kg) values compared with the reference value (18 kg): 6.84 (2.84–16.47) and 0.06 (0.02–0.21), respectively] risk even after adjusting for FMI and SMI (*Figure* 3F and 3H).

**Figure 4:**
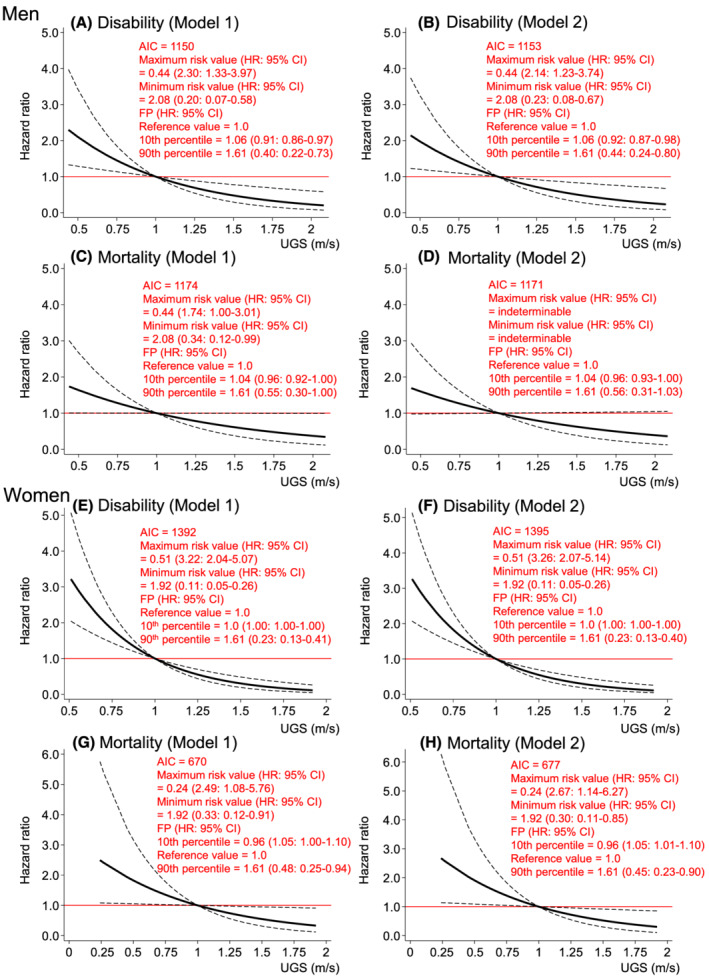
Dose–response relationships of UGS with incident disability and mortality risk. *Figure* 4A–4D shows the relationships of UGS with disability (*Figure* 4A–4B) and mortality (*Figure* 4C–4D) risks in men. *Figure* 4E–4H shows the relationships of UGS with disability (*Figure* 4E–4F) and mortality (*Figure* 4G–4H) risks in women. Figure 4A–4H was modelled using an FP function. Model 1 was adjusted for baseline age, study area, year of first visit for health check‐up, drinking and smoking status, hypertension, stroke, heart disease, diabetes, cancer, high total cholesterol, low total cholesterol, hypoalbuminemia, anaemia, chronic kidney disease, low activity, depressed mood, and cognitive impairment. Model 2 was adjusted for the variables in Model 1, plus FMI and SMI. The reference values for each model are the cut‐off points for sarcopenia criteria defined by the Asian Working Group for Sarcopenia in 2019 (i.e. UGS of 1.0 m/s in both sexes). The dashed lines indicate the 95% confidence intervals. AIC, Akaike's information criterion; FMI, fat mass index; FP, fractional polynomial; HR, hazard ratio; SMI, skeletal muscle mass index; UGS, usual gait speed.

*Figure* 4 shows the dose–response relationships of UGS with incident disability and mortality risks. UGS consistently showed a significant inverse dose–response relationship with disability in both sexes [HRs (95% CIs) of minimum and maximum values compared with the reference value (1.0 m/s): 2.14 (1.23–3.74) and 0.23 (0.08–0.67), respectively, in men (*Figure* 4B); 3.26 (2.07–5.14) and 0.11 (0.05–0.26), respectively, in women (*Figure* 4F)]. Although UGS in women also showed a significant inverse dose–response relationship with mortality even after further adjustment for FMI and SMI [HRs (95% CIs) of minimum and maximum values compared with the reference value (1.0 m/s): 2.67 (1.14–6.27) and 0.30 (0.11–0.85), respectively, in women (*Figure* 4H)], the significant UGS–mortality association disappeared in men (*Figure* 4D).

Results of the sensitivity analyses that excluded disability or death that occurred during the first year of follow‐up were not substantially different from those of the primary analyses for both sexes (Supporting Information, *Figures* S1, S2, S3, & S4). In the analyses that excluded disability or death that occurred during the first 2 years, although the associations of SMI, HGS, and UGS with both outcomes were weakened in men, other results were not substantially different from those of the primary analyses (*Figures* S5, S6, S7, & S8).

## Discussion

This sex‐stratified multivariate dose–response analysis showed that FMI and SMI did not significantly impact disability risk, while HGS and UGS consistently exhibited an inverse dose–response relationship with disability in both sexes, independent of body composition. Conversely, sex‐related differences were observed in the association of each parameter with mortality risk. In men, the SMI–mortality association remained significant even after HGS and UGS adjustments, whereas HGS– and UGS–mortality associations disappeared after body composition adjustment. FMI had no impact on mortality risk. In women, HGS and UGS consistently exhibited a clear inverse dose–response relationship with disability and mortality, independent of body composition. Although SMI had no impact on mortality risk, a lower FMI was significantly associated with a higher mortality risk, independent of HGS and UGS.

We previously reported that fat‐free mass index and SMI were more definitive predictors of mortality than BMI and FMI.^21^ The results of men in the current study were consistent with those of this previous study.^21^ However, in the current study, different results were observed in which the SMI–mortality association disappeared, and a lower FMI affected mortality risk in women. These discrepancies are attributable to adjustment for important covariates that have a strong impact on disability and mortality (i.e. low activity, depressive symptoms, cognitive impairment, HGS, and UGS) in addition to the covariates of our previous study.^21^ Therefore, the results of the current study are considered more reasonable than that of our previous study.

Our results in women were consistent with those of previous studies that showed that muscle strength and physical performance are more strongly associated with health‐related outcomes than muscle mass.^11^, ^12^, ^18^ We provided further evidence that a lower FMI increases mortality risk, independent of HGS and UGS. Moreover, a noteworthy finding of this study was that in men, the disability risk was more dependent on muscle quality (i.e. HGS), while mortality risk was more dependent on muscle quantity (i.e. SMI). The annual rate of muscle strength decline has been reported to be approximately three times greater than the rates of concomitant loss of muscle mass, which is more pronounced in men than in women.^16^ Therefore, early decline in muscle strength may have a stronger impact on disability, which is an outcome that presents earlier than mortality in general, and muscle mass may be independently associated with mortality in men.

The SMI–mortality association, independent of HGS, has also been reported in a recent systematic review and meta‐analysis.^37^ Muscle mass is a crucial reservoir of amino acids and effector molecules, such as myokines and cytokines, which help in combating illness, infection, and wasting.^38^ Therefore, it may be associated with a wide range of life‐threatening adverse health effects, especially in older adults.^38^ Moreover, our results may be attributable to sex‐related differences in muscle mass and fat mass. Men had a greater muscle mass and wider distribution than women, whereas women had a greater fat mass and wider distribution than men. This may provide an opportunity to better capture the heterogeneous risk profiles of individuals. Specifically, older Japanese men and women in this study had substantially lower FMI and higher SMI than those in previously studied Western populations.^21^ These findings may explain the significant impact of SMI in men and a lower FMI in women on mortality risk.

There are some limitations of this study. First, selection bias is a concern because our study participants were limited to individuals who had undergone check‐ups. Second, women had a low mortality rate, and we could not analyse the association of sarcopenia parameters with cause‐specific disability and mortality. Third, in our follow‐up period, the possible influence of reverse causation cannot be completely excluded, compared with previous studies with long‐term follow‐up.^35^ However, we believe that a 5.3‐year follow‐up period is reasonable because predictive ability declines over time.^39^, ^40^ Fourth, although we statistically adjusted many covariates, there may be factors that we have not considered, such as polypharmacy and potentially inappropriate medications, which affect sarcopenia and disability in older adults.^41^ Fifth, although HGS is a simple and useful index of muscle strength, it should not be used as a sole measure of overall muscle strength, especially in men.^42^ More plausible conclusions regarding the strength–mortality association in men may be drawn by defining muscle strength as quadriceps strength in future studies. Moreover, the low activity variable we used may be insufficient to adjust for the confounding effects of physical activity. Nevertheless, we believe that our results would not differ significantly even after adjusting for physical activity, because a previous study reported significant associations of low muscle strength with increased risk of all‐cause mortality, independent of sedentary time and leisure‐time physical activity.^18^ Finally, although we did confirm that direct segmental multi‐frequency bioelectrical impedance analysis has an acceptable accuracy,^25^ it probably underestimated lean mass and overestimated fat mass among older Japanese individuals compared with the measurements with dual‐energy X‐ray absorptiometry. Systematic bias is another concern when comparing measured dual‐energy X‐ray absorptiometry values.

Despite these limitations, to our knowledge, this is the first study to report on the shapes of the associations of body composition, muscle strength, and physical performance with incident disability and mortality risks simultaneously. Using spline analyses rather than a categorical or linear approach, we were able to continuously show the HRs for disability and mortality of each measurement (*Figures* 1, 2, 3, 4). We believe that this information is useful for screening high‐risk individuals in clinical and public health settings. It should be noted that the associations of each measurement with disability and mortality risks are not necessarily linear, as depicted in these results.

In conclusion, disability risk was consistently more dependent on muscle strength (defined by HGS) and physical performance (defined by UGS) than body composition in both sexes, while mortality risk was also influenced by body composition, independent of muscle strength and physical performance. In particular, mortality risk in men was more dependent on muscle mass than muscle strength and physical performance, and mortality risk in women was influenced by lower fat mass along with muscle strength and physical performance. Although improving muscle strength and physical performance (rather than only increasing muscle mass) should be the first target for health promotion, it is also necessary to pay attention to body composition to extend life expectancy in older adults.

## Conflict of interest

The authors declare that they have no competing interests.

## Funding

This study was supported by grants from the Tokyo Metropolitan Institute of Gerontology; Research Institute of Science and Technology for Society, the Japan Science and Technology Agency; Grants‐In‐Aid for Scientific Research (B) JP20390190, (B) JP21390212, (B) JP24390173, and (B) JP26310111 from the Ministry of Education, Culture, Sports, Science and Technology, Japan; the Japan Arteriosclerosis Prevention Fund for the Japan Arteriosclerosis Longitudinal Study (2001–2012); and the towns of Kusatsu and Hatoyama.

## Supporting information


**Figure S1.**Dose–response relationships of FMI with incident disability and mortality risks, excluding disabilities or deaths that occurred during the first year of follow‐up
**Figure S1a‐S1d** show the relationships of FMI with disability (Figure S1a‐S1b) and mortality (Figure S1c‐S1d) risks in men. Figure S1e‐S1h show the relationships between FMI and disability (Figure S1e‐S1f) and mortality (Figure S1g‐S1h) risks in women. Figure S1a‐S1b were modeled using an FP function, and Figure S1c‐S1h were modeled using an RCS with three knots located at the 10^th^, 50^th^, and 90^th^ percentiles of the distribution of the index. Model 1 was adjusted for baseline age, study area, year of first visit for health check‐up, drinking and smoking status, hypertension, stroke, heart disease, diabetes, cancer, high total cholesterol, low total cholesterol, hypoalbuminemia, anemia, chronic kidney disease, low activity, depressed mood, cognitive impairment, and SMI. Model 2 was adjusted for the variables in Model 1 plus HGS and UGS. The reference values for each model are the median FMI (i.e., FMI of 5.7 kg/m^2^ in men and FMI of 7.4 kg/m^2^ in women). The dashed lines indicate the 95% confidence intervals. AIC, Akaike's information criterion; FMI, fat mass index; FP, fractional polynomial; HGS, handgrip strength; HR, hazard ratio; RCS, restricted cubic spline; SMI, skeletal muscle mass index; UGS, usual gait speed.

**Figure S2.** Dose–response relationships of SMI with incident disability and mortality risks, excluding disabilities or deaths that occurred during the first year of follow‐up
**Figure S2a‐S2d** show the relationships of SMI with disability (Figure S2a‐S2b) and mortality (Figure S2c‐S2d) risks in men. Figure S2e‐S2h show the relationships between SMI and disability (Figure S2e‐S2f) and mortality (Figure S2g‐S2h) risks in women. Figure S2a‐S2h were modeled using an FP function. Model 1 was adjusted for baseline age, study area, year of first visit for health check‐up, alcohol consumption and smoking status, hypertension, stroke, heart disease, diabetes, cancer, high total cholesterol, low total cholesterol, hypoalbuminemia, anemia, chronic kidney disease, low activity, depressed mood, cognitive impairment, and FMI. Model 2 was adjusted for the variables in Model 1 plus HGS and UGS. The reference values for each model are the cut‐off points for sarcopenia criteria defined by the Asian Working Group for Sarcopenia in 2019 (i.e., SMI of 7.0 kg/m^2^ in men and SMI of 5.7 kg/m^2^ in women). The dashed lines indicate the 95% confidence intervals. AIC, Akaike's information criterion; FMI, fat mass index; FP, fractional polynomial; HGS, handgrip strength; HR, hazard ratio; SMI, skeletal muscle mass index; UGS, usual gait speed.

**Figure S3.** Dose–response relationships of HGS with incident disability and mortality risks, excluding disabilities or deaths that occurred during the first year of follow‐up
**Figure S3a‐S3d** show the relationships of HGS with disability (Figure S3a‐S3b) and mortality (Figure S3c‐S3d) risks in men. Figure S3e‐S3h show the relationships of HGS with disability (Figure S3e‐S3f) and mortality (Figure S3g‐S3h) risks in women. Figure S3a‐S3h were modeled using an FP function. Model 1 was adjusted for baseline age, study area, year of first visit for health check‐up, drinking and smoking status, hypertension, stroke, heart disease, diabetes, cancer, high total cholesterol, low total cholesterol, hypoalbuminemia, anemia, chronic kidney disease, low activity, depressed mood, and cognitive impairment. Model 2 was adjusted for the variables in Model 1 plus FMI and SMI. The reference values for each model are the cut‐off points for sarcopenia criteria defined by the Asian Working Group for Sarcopenia in 2019 (i.e., HGS of 28 kg in men and HGS of 18 kg in women). The dashed lines indicate the 95% confidence intervals. AIC, Akaike's information criterion; FMI, fat mass index; FP, fractional polynomial; HGS, handgrip strength; HR, hazard ratio; SMI, skeletal muscle mass index.

**Figure S4.** Dose–response relationships of UGS with incident disability and mortality risks, excluding disabilities or deaths that occurred during the first year of follow‐up
**Figure S4a‐S4d** show the relationships of UGS with disability (Figure S4a‐S4b) and mortality (Figure S4c‐S4d) risks in men. Figure S4e‐S4h show the relationships of UGS with disability (Figure S4e‐S4f) and mortality (Figure S4g‐S4h) risks in women. Figure S4a‐S4h were modeled using an FP function. Model 1 was adjusted for baseline age, study area, year of first visit for health check‐up, drinking and smoking status, hypertension, stroke, heart disease, diabetes, cancer, high total cholesterol, low total cholesterol, hypoalbuminemia, anemia, chronic kidney disease, low activity, depressed mood, and cognitive impairment. Model 2 was adjusted for the variables in Model 1 plus FMI and SMI. The reference values for each model are the cut‐off points for sarcopenia criteria defined by the Asian Working Group for Sarcopenia in 2019 (i.e., UGS of 1.0 m/s in both sexes). The dashed lines indicate the 95% confidence intervals. AIC, Akaike's information criterion; FMI, fat mass index; FP, fractional polynomial; HR, hazard ratio; SMI, skeletal muscle mass index; UGS, usual gait speed.

**Figure S5.** Dose–response relationships of FMI with incident disability and mortality risks, excluding disabilities or deaths that occurred during the first two years of follow‐up
**Figure S5a‐S5d** show the relationships of FMI with disability (Figure S5a‐S5b) and mortality (Figure S5c‐S5d) risks in men. Figure S5e‐S5h show the relationships of FMI with disability (Figure S5e‐S5f) and mortality (Figure S5g‐S5h) risks in women. Figure S5a‐S5b were modeled using an FP function, and Figure S5c‐S5h were modeled using an RCS with three knots located at the 10^th^, 50^th^, and 90^th^ percentiles of the distribution of the index. Model 1 was adjusted for baseline age, study area, year of first visit for health check‐up, drinking and smoking status, hypertension, stroke, heart disease, diabetes, cancer, high total cholesterol, low total cholesterol, hypoalbuminemia, anemia, chronic kidney disease, low activity, depressed mood, cognitive impairment, and SMI. Model 2 was adjusted for the variables in Model 1 plus HGS and UGS. The reference values for each model are the median FMI (i.e., FMI of 5.7 kg/m^2^ in men and FMI of 7.4 kg/m^2^ in women). The dashed lines indicate the 95% confidence intervals. AIC, Akaike's information criterion; FMI, fat mass index; FP, fractional polynomial; HGS, handgrip strength; HR, hazard ratio; RCS, restricted cubic spline; SMI, skeletal muscle mass index; UGS, usual gait speed.

**Figure S6.** Dose–response relationships of SMI with incident disability and mortality risks, excluding disabilities or deaths that occurred during the first two years of follow‐up
**Figure S6a‐S6d** show the relationships of SMI with disability (Figure S6a‐S6b) and mortality (Figure S6c‐S6d) risks in men. Figure S6e‐S6h show the relationships of SMI with disability (Figure S6e‐S6f) and mortality (Figure S6g‐S6h) risks in women. Figure S6a‐S6h were modeled using an FP function. Model 1 was adjusted for baseline age, study area, year of first visit for health check‐up, alcohol consumption and smoking status, hypertension, stroke, heart disease, diabetes, cancer, high total cholesterol, low total cholesterol, hypoalbuminemia, anemia, chronic kidney disease, low activity, depressed mood, cognitive impairment, and FMI. Model 2 was adjusted for the variables in Model 1 plus HGS and UGS. The reference values for each model are the cut‐off points for sarcopenia criteria defined by the Asian Working Group for Sarcopenia in 2019 (i.e., SMI of 7.0 kg/m^2^ in men and SMI of 5.7 kg/m^2^ in women). The dashed lines indicate the 95% confidence intervals. AIC, Akaike's information criterion; FMI, fat mass index; FP, fractional polynomial; HGS, handgrip strength; HR, hazard ratio; SMI, skeletal muscle mass index; UGS, usual gait speed.

**Figure S7.** Dose–response relationships of HGS with incident disability and mortality risks, excluding disabilities or deaths that occurred during the first two years of follow‐up
**Figure S7a‐S7d** show the relationships of HGS with disability (Figure S7a‐S7b) and mortality (Figure S7c‐S7d) risks in men. Figure S7e‐S7h show the relationships of HGS with disability (Figure S7e‐S7f) and mortality (Figure S7g‐S7h) risks in women. Figure S7a‐S7h were modeled using an FP function. Model 1 was adjusted for baseline age, study area, year of first visit for health check‐up, drinking and smoking status, hypertension, stroke, heart disease, diabetes, cancer, high total cholesterol, low total cholesterol, hypoalbuminemia, anemia, chronic kidney disease, low activity, depressed mood, and cognitive impairment. Model 2 was adjusted for the variables in Model 1 plus FMI and SMI. The reference values for each model are the cut‐off points for sarcopenia criteria defined by the Asian Working Group for Sarcopenia in 2019 (i.e., HGS of 28 kg in men and HGS of 18 kg in women). The dashed lines indicate the 95% confidence intervals. AIC, Akaike's information criterion; FMI, fat mass index; FP, fractional polynomial; HGS, handgrip strength; HR, hazard ratio; SMI, skeletal muscle mass index.

**Figure S8.** Dose–response relationships of UGS with incident disability and mortality risks, excluding disabilities or deaths that occurred during the first two years of follow‐up
**Figure S8a‐S8d** show the relationships of UGS with disability (Figure S8a‐S8b) and mortality (Figure S8c‐S8d) risks in men. Figure S8e‐S8h show the relationships of UGS with disability (Figure S8e‐S8f) and mortality (Figure S8g‐S8h) risks in women. Figure S8a‐S8h were modeled using an FP function. Model 1 was adjusted for baseline age, study area, year of first visit for health check‐up, drinking and smoking status, hypertension, stroke, heart disease, diabetes, cancer, high total cholesterol, low total cholesterol, hypoalbuminemia, anemia, chronic kidney disease, low activity, depressed mood, and cognitive impairment. Model 2 was adjusted for the variables in Model 1 plus FMI and SMI. The reference values for each model are the cut‐off points for sarcopenia criteria defined by the Asian Working Group for Sarcopenia in 2019 (i.e., UGS of 1.0 m/s in both sexes). The dashed lines indicate the 95% confidence intervals. AIC, Akaike's information criterion; FMI, fat mass index; FP, fractional polynomial; HR, hazard ratio; SMI, skeletal muscle mass index; UGS, usual gait speed.
