# Supplementary material for: Dose–response relationships of sarcopenia parameters with incident disability and mortality in older Japanese adults
Source: J Cachexia Sarcopenia Muscle. 2022 Feb 25;13(2):932–44. doi: 10.1002/jcsm.12958 (PMC8977959; doi:10.1002/jcsm.12958)
Supplement: Supplementary file 1 — Figure S1.Dose–response relationships of FMI with incident disability and mortality risks, excluding disabilities or deaths that occurred during the first year of follow‐up Figure S1a‐S1d show the relationships of FMI with disability (Figure S1a‐S1b) and mortality (Figure S1c‐S1d) risks in men. Figure S1e‐S1h show the relationships between FMI and disability (Figure S1e‐S1f) and mortality (Figure S1g‐S1h) risks in women. Figure S1a‐S1b were modeled using an FP function, and Figure S1c‐S1h were modeled using an RCS with three knots located at the 10th, 50th, and 90th percentiles of the distribution of the index. Model 1 was adjusted for baseline age, study area, year of first visit for health check‐up, drinking and smoking status, hypertension, stroke, heart disease, diabetes, cancer, high total cholesterol, low total cholesterol, hypoalbuminemia, anemia, chronic kidney disease, low activity, depressed mood, cognitive impairment, and SMI. Model 2 was adjusted for the variables in Model 1 plus HGS and UGS. The reference values for each model are the median FMI (i.e., FMI of 5.7 kg/m2 in men and FMI of 7.4 kg/m2 in women). The dashed lines indicate the 95% confidence intervals. AIC, Akaike's information criterion; FMI, fat mass index; FP, fractional polynomial; HGS, handgrip strength; HR, hazard ratio; RCS, restricted cubic spline; SMI, skeletal muscle mass index; UGS, usual gait speed. [file JCSM-13-932-s002.pptx]

## Slide 1
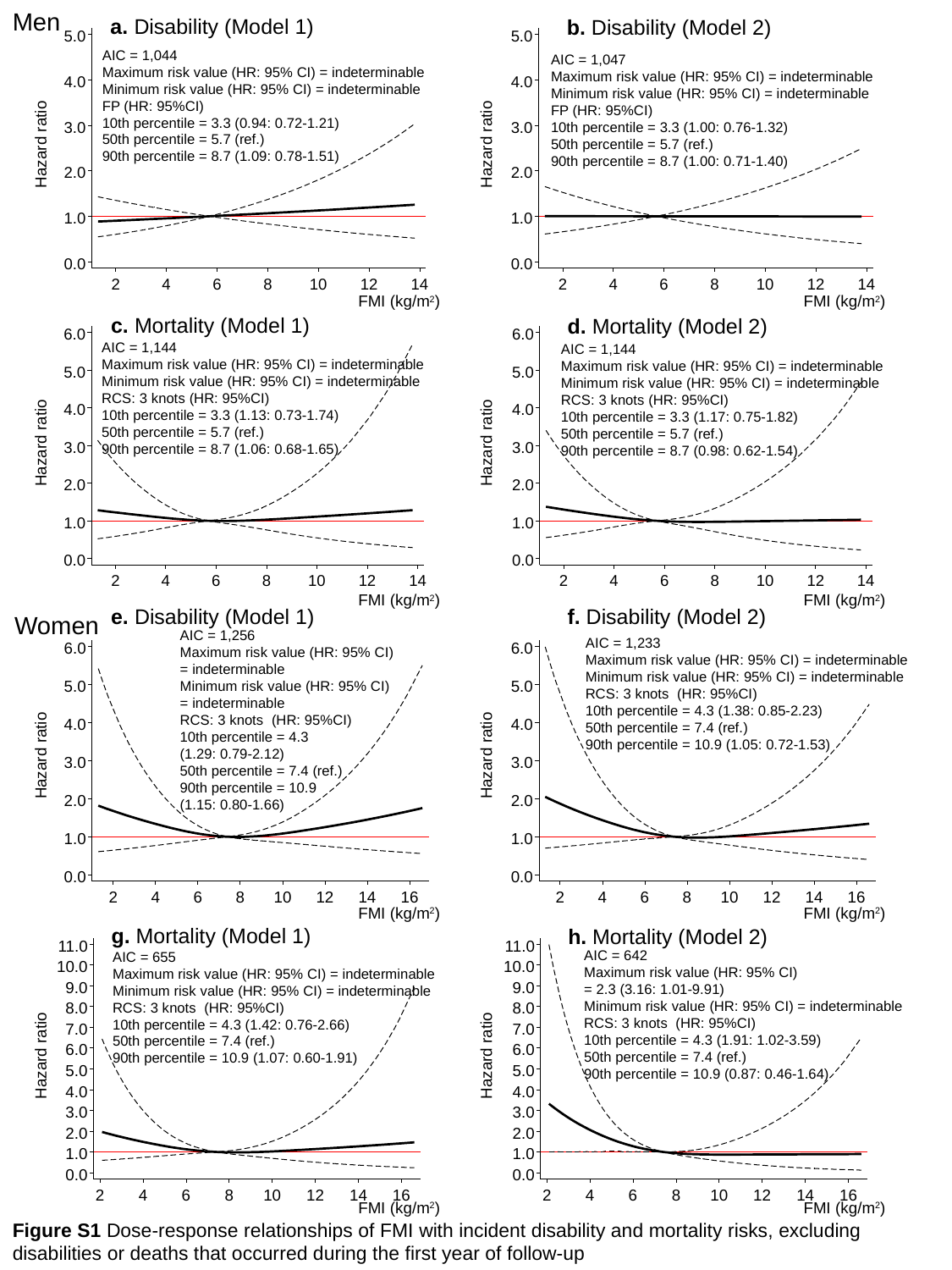

Men
a. Disability (Model 1)
b. Disability (Model 2)
AIC = 1,044
Maximum risk value (HR: 95% CI) = indeterminable
Minimum risk value (HR: 95% CI) = indeterminable
FP (HR: 95%CI)
10th percentile = 3.3 (0.94: 0.72-1.21)
50th percentile = 5.7 (ref.)
90th percentile = 8.7 (1.09: 0.78-1.51)
AIC = 1,047
Maximum risk value (HR: 95% CI) = indeterminable
Minimum risk value (HR: 95% CI) = indeterminable
FP (HR: 95%CI)
10th percentile = 3.3 (1.00: 0.76-1.32)
50th percentile = 5.7 (ref.)
90th percentile = 8.7 (1.00: 0.71-1.40)
Hazard ratio
Hazard ratio
FMI (kg/m2)
FMI (kg/m2)
c. Mortality (Model 1)
d. Mortality (Model 2)
AIC = 1,144
Maximum risk value (HR: 95% CI) = indeterminable
Minimum risk value (HR: 95% CI) = indeterminable
RCS: 3 knots (HR: 95%CI)
10th percentile = 3.3 (1.13: 0.73-1.74)
50th percentile = 5.7 (ref.)
90th percentile = 8.7 (1.06: 0.68-1.65)
AIC = 1,144
Maximum risk value (HR: 95% CI) = indeterminable
Minimum risk value (HR: 95% CI) = indeterminable
RCS: 3 knots (HR: 95%CI)
10th percentile = 3.3 (1.17: 0.75-1.82)
50th percentile = 5.7 (ref.)
90th percentile = 8.7 (0.98: 0.62-1.54)
Hazard ratio
Hazard ratio
FMI (kg/m2)
FMI (kg/m2)
e. Disability (Model 1)
f. Disability (Model 2)
Women
AIC = 1,256
Maximum risk value (HR: 95% CI)
= indeterminable
Minimum risk value (HR: 95% CI)
= indeterminable
RCS: 3 knots (HR: 95%CI)
10th percentile = 4.3
(1.29: 0.79-2.12)
50th percentile = 7.4 (ref.)
90th percentile = 10.9
(1.15: 0.80-1.66)
AIC = 1,233
Maximum risk value (HR: 95% CI) = indeterminable
Minimum risk value (HR: 95% CI) = indeterminable
RCS: 3 knots (HR: 95%CI)
10th percentile = 4.3 (1.38: 0.85-2.23)
50th percentile = 7.4 (ref.)
90th percentile = 10.9 (1.05: 0.72-1.53)
Hazard ratio
Hazard ratio
FMI (kg/m2)
FMI (kg/m2)
g. Mortality (Model 1)
h. Mortality (Model 2)
AIC = 642
Maximum risk value (HR: 95% CI)
= 2.3 (3.16: 1.01-9.91)
Minimum risk value (HR: 95% CI) = indeterminable
RCS: 3 knots (HR: 95%CI)
10th percentile = 4.3 (1.91: 1.02-3.59)
50th percentile = 7.4 (ref.)
90th percentile = 10.9 (0.87: 0.46-1.64)
AIC = 655
Maximum risk value (HR: 95% CI) = indeterminable
Minimum risk value (HR: 95% CI) = indeterminable
RCS: 3 knots (HR: 95%CI)
10th percentile = 4.3 (1.42: 0.76-2.66)
50th percentile = 7.4 (ref.)
90th percentile = 10.9 (1.07: 0.60-1.91)
Hazard ratio
Hazard ratio
FMI (kg/m2)
FMI (kg/m2)
Figure S1 Dose-response relationships of FMI with incident disability and mortality risks, excluding disabilities or deaths that occurred during the first year of follow-up
