# Supplementary material for: Dose–response relationships of sarcopenia parameters with incident disability and mortality in older Japanese adults
Source: J Cachexia Sarcopenia Muscle. 2022 Feb 25;13(2):932–44. doi: 10.1002/jcsm.12958 (PMC8977959; doi:10.1002/jcsm.12958)
Supplement: Supplementary file 6 — Figure S6. Dose–response relationships of SMI with incident disability and mortality risks, excluding disabilities or deaths that occurred during the first two years of follow‐up Figure S6a‐S6d show the relationships of SMI with disability (Figure S6a‐S6b) and mortality (Figure S6c‐S6d) risks in men. Figure S6e‐S6h show the relationships of SMI with disability (Figure S6e‐S6f) and mortality (Figure S6g‐S6h) risks in women. Figure S6a‐S6h were modeled using an FP function. Model 1 was adjusted for baseline age, study area, year of first visit for health check‐up, alcohol consumption and smoking status, hypertension, stroke, heart disease, diabetes, cancer, high total cholesterol, low total cholesterol, hypoalbuminemia, anemia, chronic kidney disease, low activity, depressed mood, cognitive impairment, and FMI. Model 2 was adjusted for the variables in Model 1 plus HGS and UGS. The reference values for each model are the cut‐off points for sarcopenia criteria defined by the Asian Working Group for Sarcopenia in 2019 (i.e., SMI of 7.0 kg/m2 in men and SMI of 5.7 kg/m2 in women). The dashed lines indicate the 95% confidence intervals. AIC, Akaike's information criterion; FMI, fat mass index; FP, fractional polynomial; HGS, handgrip strength; HR, hazard ratio; SMI, skeletal muscle mass index; UGS, usual gait speed. [file JCSM-13-932-s001.pptx]

## Slide 1
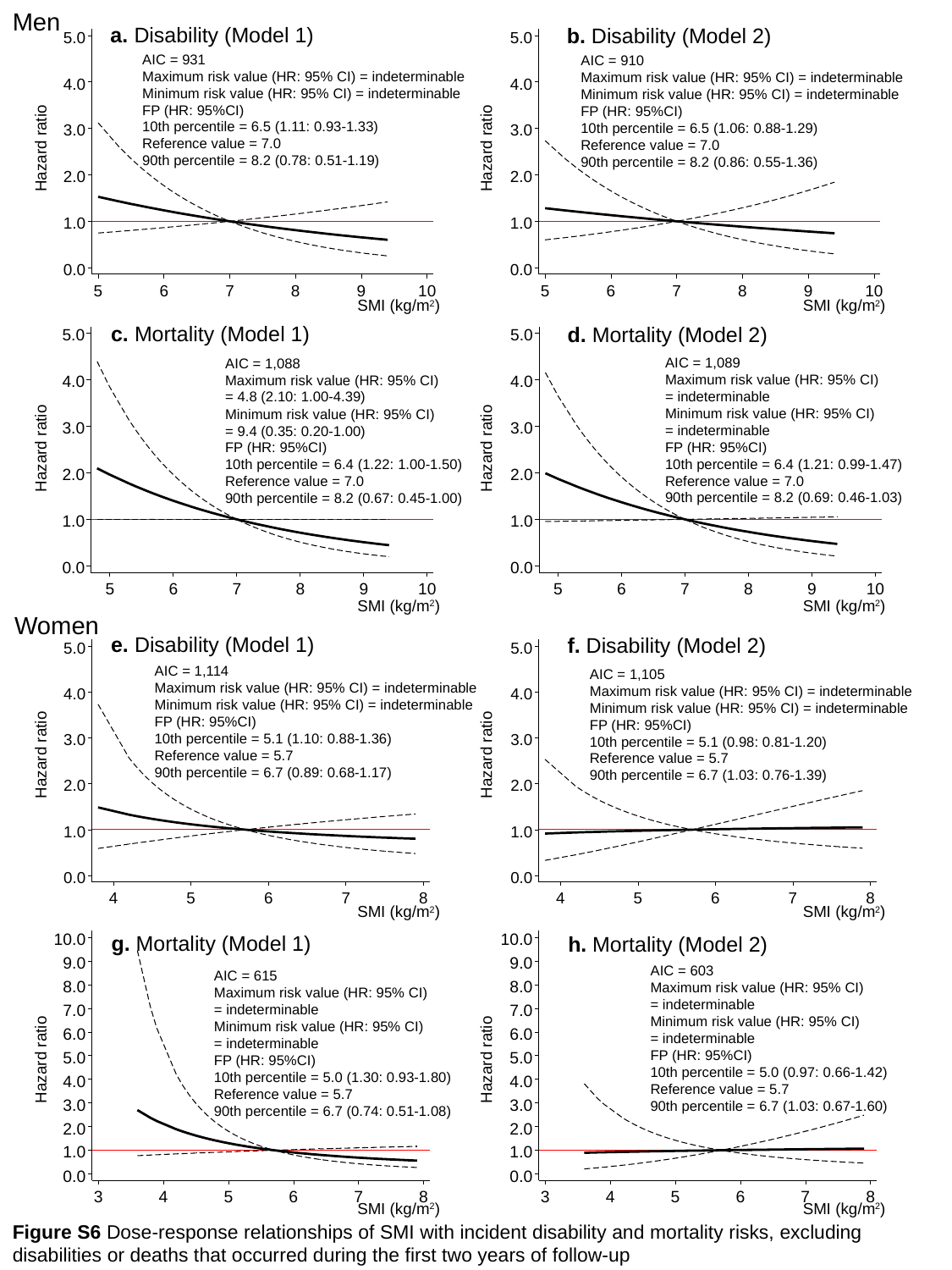

Men
a. Disability (Model 1)
b. Disability (Model 2)
AIC = 931
Maximum risk value (HR: 95% CI) = indeterminable
Minimum risk value (HR: 95% CI) = indeterminable
FP (HR: 95%CI)
10th percentile = 6.5 (1.11: 0.93-1.33)
Reference value = 7.0
90th percentile = 8.2 (0.78: 0.51-1.19)
AIC = 910
Maximum risk value (HR: 95% CI) = indeterminable
Minimum risk value (HR: 95% CI) = indeterminable
FP (HR: 95%CI)
10th percentile = 6.5 (1.06: 0.88-1.29)
Reference value = 7.0
90th percentile = 8.2 (0.86: 0.55-1.36)
Hazard ratio
Hazard ratio
SMI (kg/m2)
SMI (kg/m2)
c. Mortality (Model 1)
d. Mortality (Model 2)
AIC = 1,089
Maximum risk value (HR: 95% CI)
= indeterminable
Minimum risk value (HR: 95% CI)
= indeterminable
FP (HR: 95%CI)
10th percentile = 6.4 (1.21: 0.99-1.47)
Reference value = 7.0
90th percentile = 8.2 (0.69: 0.46-1.03)
AIC = 1,088
Maximum risk value (HR: 95% CI)
= 4.8 (2.10: 1.00-4.39)
Minimum risk value (HR: 95% CI)
= 9.4 (0.35: 0.20-1.00)
FP (HR: 95%CI)
10th percentile = 6.4 (1.22: 1.00-1.50)
Reference value = 7.0
90th percentile = 8.2 (0.67: 0.45-1.00)
Hazard ratio
Hazard ratio
SMI (kg/m2)
SMI (kg/m2)
Women
e. Disability (Model 1)
f. Disability (Model 2)
AIC = 1,114
Maximum risk value (HR: 95% CI) = indeterminable
Minimum risk value (HR: 95% CI) = indeterminable
FP (HR: 95%CI)
10th percentile = 5.1 (1.10: 0.88-1.36)
Reference value = 5.7
90th percentile = 6.7 (0.89: 0.68-1.17)
AIC = 1,105
Maximum risk value (HR: 95% CI) = indeterminable
Minimum risk value (HR: 95% CI) = indeterminable
FP (HR: 95%CI)
10th percentile = 5.1 (0.98: 0.81-1.20)
Reference value = 5.7
90th percentile = 6.7 (1.03: 0.76-1.39)
Hazard ratio
Hazard ratio
SMI (kg/m2)
SMI (kg/m2)
g. Mortality (Model 1)
h. Mortality (Model 2)
AIC = 603
Maximum risk value (HR: 95% CI)
= indeterminable
Minimum risk value (HR: 95% CI)
= indeterminable
FP (HR: 95%CI)
10th percentile = 5.0 (0.97: 0.66-1.42)
Reference value = 5.7
90th percentile = 6.7 (1.03: 0.67-1.60)
AIC = 615
Maximum risk value (HR: 95% CI)
= indeterminable
Minimum risk value (HR: 95% CI)
= indeterminable
FP (HR: 95%CI)
10th percentile = 5.0 (1.30: 0.93-1.80)
Reference value = 5.7
90th percentile = 6.7 (0.74: 0.51-1.08)
Hazard ratio
Hazard ratio
SMI (kg/m2)
SMI (kg/m2)
Figure S6 Dose-response relationships of SMI with incident disability and mortality risks, excluding disabilities or deaths that occurred during the first two years of follow-up
