# Supplementary material for: Dose–response relationships of sarcopenia parameters with incident disability and mortality in older Japanese adults
Source: J Cachexia Sarcopenia Muscle. 2022 Feb 25;13(2):932–44. doi: 10.1002/jcsm.12958 (PMC8977959; doi:10.1002/jcsm.12958)
Supplement: Supplementary file 7 — Figure S7. Dose–response relationships of HGS with incident disability and mortality risks, excluding disabilities or deaths that occurred during the first two years of follow‐up Figure S7a‐S7d show the relationships of HGS with disability (Figure S7a‐S7b) and mortality (Figure S7c‐S7d) risks in men. Figure S7e‐S7h show the relationships of HGS with disability (Figure S7e‐S7f) and mortality (Figure S7g‐S7h) risks in women. Figure S7a‐S7h were modeled using an FP function. Model 1 was adjusted for baseline age, study area, year of first visit for health check‐up, drinking and smoking status, hypertension, stroke, heart disease, diabetes, cancer, high total cholesterol, low total cholesterol, hypoalbuminemia, anemia, chronic kidney disease, low activity, depressed mood, and cognitive impairment. Model 2 was adjusted for the variables in Model 1 plus FMI and SMI. The reference values for each model are the cut‐off points for sarcopenia criteria defined by the Asian Working Group for Sarcopenia in 2019 (i.e., HGS of 28 kg in men and HGS of 18 kg in women). The dashed lines indicate the 95% confidence intervals. AIC, Akaike's information criterion; FMI, fat mass index; FP, fractional polynomial; HGS, handgrip strength; HR, hazard ratio; SMI, skeletal muscle mass index. [file JCSM-13-932-s008.pptx]

## Slide 1
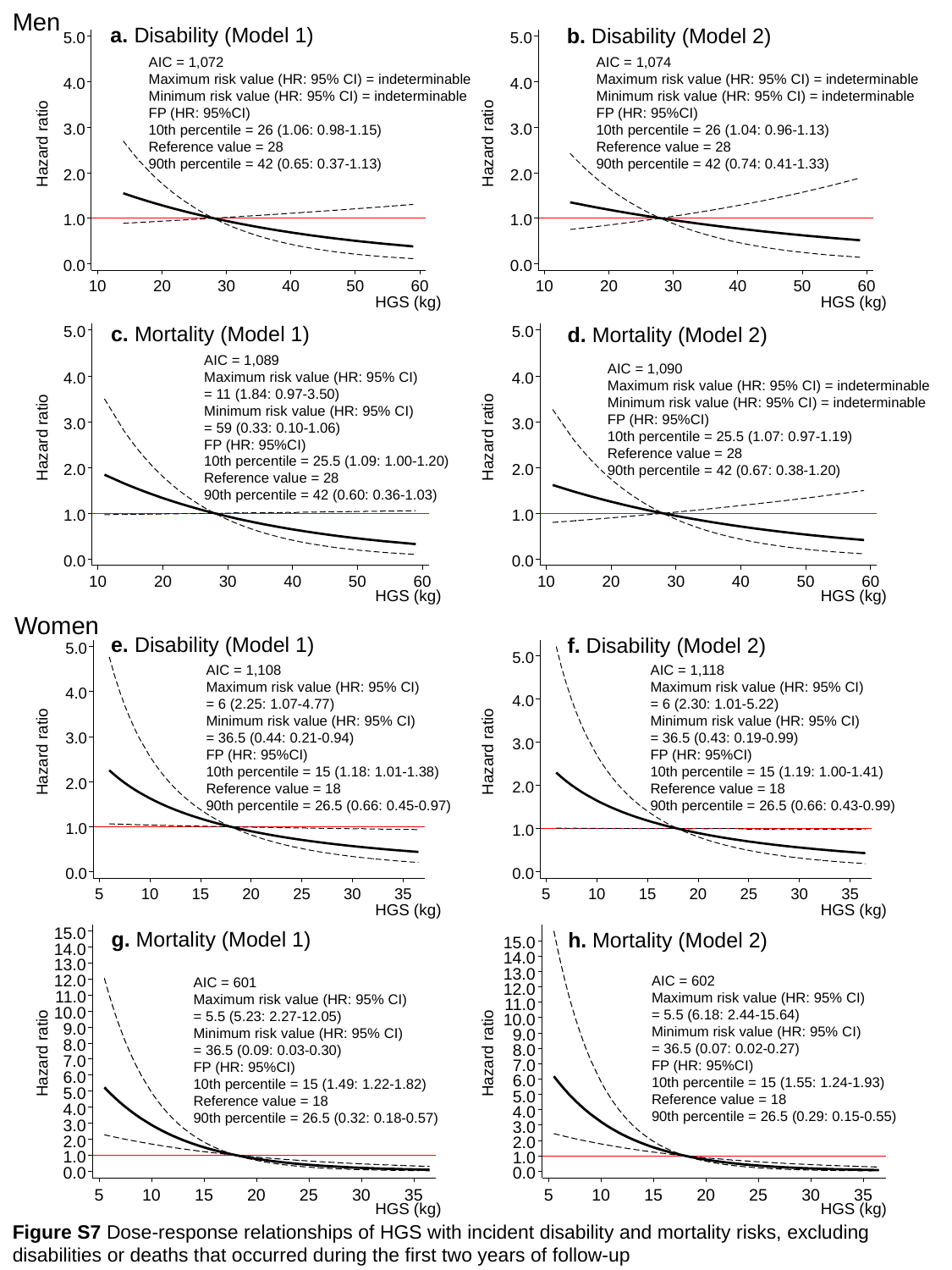

Men
a. Disability (Model 1)
b. Disability (Model 2)
AIC = 1,072
Maximum risk value (HR: 95% CI) = indeterminable
Minimum risk value (HR: 95% CI) = indeterminable
FP (HR: 95%CI)
10th percentile = 26 (1.06: 0.98-1.15)
Reference value = 28
90th percentile = 42 (0.65: 0.37-1.13)
AIC = 1,074
Maximum risk value (HR: 95% CI) = indeterminable
Minimum risk value (HR: 95% CI) = indeterminable
FP (HR: 95%CI)
10th percentile = 26 (1.04: 0.96-1.13)
Reference value = 28
90th percentile = 42 (0.74: 0.41-1.33)
Hazard ratio
Hazard ratio
HGS (kg)
HGS (kg)
c. Mortality (Model 1)
d. Mortality (Model 2)
AIC = 1,089
Maximum risk value (HR: 95% CI)
= 11 (1.84: 0.97-3.50)
Minimum risk value (HR: 95% CI)
= 59 (0.33: 0.10-1.06)
FP (HR: 95%CI)
10th percentile = 25.5 (1.09: 1.00-1.20)
Reference value = 28
90th percentile = 42 (0.60: 0.36-1.03)
AIC = 1,090
Maximum risk value (HR: 95% CI) = indeterminable
Minimum risk value (HR: 95% CI) = indeterminable
FP (HR: 95%CI)
10th percentile = 25.5 (1.07: 0.97-1.19)
Reference value = 28
90th percentile = 42 (0.67: 0.38-1.20)
Hazard ratio
Hazard ratio
HGS (kg)
HGS (kg)
Women
e. Disability (Model 1)
f. Disability (Model 2)
AIC = 1,108
Maximum risk value (HR: 95% CI)
= 6 (2.25: 1.07-4.77)
Minimum risk value (HR: 95% CI)
= 36.5 (0.44: 0.21-0.94)
FP (HR: 95%CI)
10th percentile = 15 (1.18: 1.01-1.38)
Reference value = 18
90th percentile = 26.5 (0.66: 0.45-0.97)
AIC = 1,118
Maximum risk value (HR: 95% CI)
= 6 (2.30: 1.01-5.22)
Minimum risk value (HR: 95% CI)
= 36.5 (0.43: 0.19-0.99)
FP (HR: 95%CI)
10th percentile = 15 (1.19: 1.00-1.41)
Reference value = 18
90th percentile = 26.5 (0.66: 0.43-0.99)
Hazard ratio
Hazard ratio
HGS (kg)
HGS (kg)
g. Mortality (Model 1)
h. Mortality (Model 2)
AIC = 602
Maximum risk value (HR: 95% CI)
= 5.5 (6.18: 2.44-15.64)
Minimum risk value (HR: 95% CI)
= 36.5 (0.07: 0.02-0.27)
FP (HR: 95%CI)
10th percentile = 15 (1.55: 1.24-1.93)
Reference value = 18
90th percentile = 26.5 (0.29: 0.15-0.55)
AIC = 601
Maximum risk value (HR: 95% CI)
= 5.5 (5.23: 2.27-12.05)
Minimum risk value (HR: 95% CI)
= 36.5 (0.09: 0.03-0.30)
FP (HR: 95%CI)
10th percentile = 15 (1.49: 1.22-1.82)
Reference value = 18
90th percentile = 26.5 (0.32: 0.18-0.57)
Hazard ratio
Hazard ratio
HGS (kg)
HGS (kg)
Figure S7 Dose-response relationships of HGS with incident disability and mortality risks, excluding disabilities or deaths that occurred during the first two years of follow-up
