# Supplementary material for: Dose–response relationships of sarcopenia parameters with incident disability and mortality in older Japanese adults
Source: J Cachexia Sarcopenia Muscle. 2022 Feb 25;13(2):932–44. doi: 10.1002/jcsm.12958 (PMC8977959; doi:10.1002/jcsm.12958)
Supplement: Supplementary file 8 — Figure S8. Dose–response relationships of UGS with incident disability and mortality risks, excluding disabilities or deaths that occurred during the first two years of follow‐up Figure S8a‐S8d show the relationships of UGS with disability (Figure S8a‐S8b) and mortality (Figure S8c‐S8d) risks in men. Figure S8e‐S8h show the relationships of UGS with disability (Figure S8e‐S8f) and mortality (Figure S8g‐S8h) risks in women. Figure S8a‐S8h were modeled using an FP function. Model 1 was adjusted for baseline age, study area, year of first visit for health check‐up, drinking and smoking status, hypertension, stroke, heart disease, diabetes, cancer, high total cholesterol, low total cholesterol, hypoalbuminemia, anemia, chronic kidney disease, low activity, depressed mood, and cognitive impairment. Model 2 was adjusted for the variables in Model 1 plus FMI and SMI. The reference values for each model are the cut‐off points for sarcopenia criteria defined by the Asian Working Group for Sarcopenia in 2019 (i.e., UGS of 1.0 m/s in both sexes). The dashed lines indicate the 95% confidence intervals. AIC, Akaike's information criterion; FMI, fat mass index; FP, fractional polynomial; HR, hazard ratio; SMI, skeletal muscle mass index; UGS, usual gait speed. [file JCSM-13-932-s007.pptx]

## Slide 1
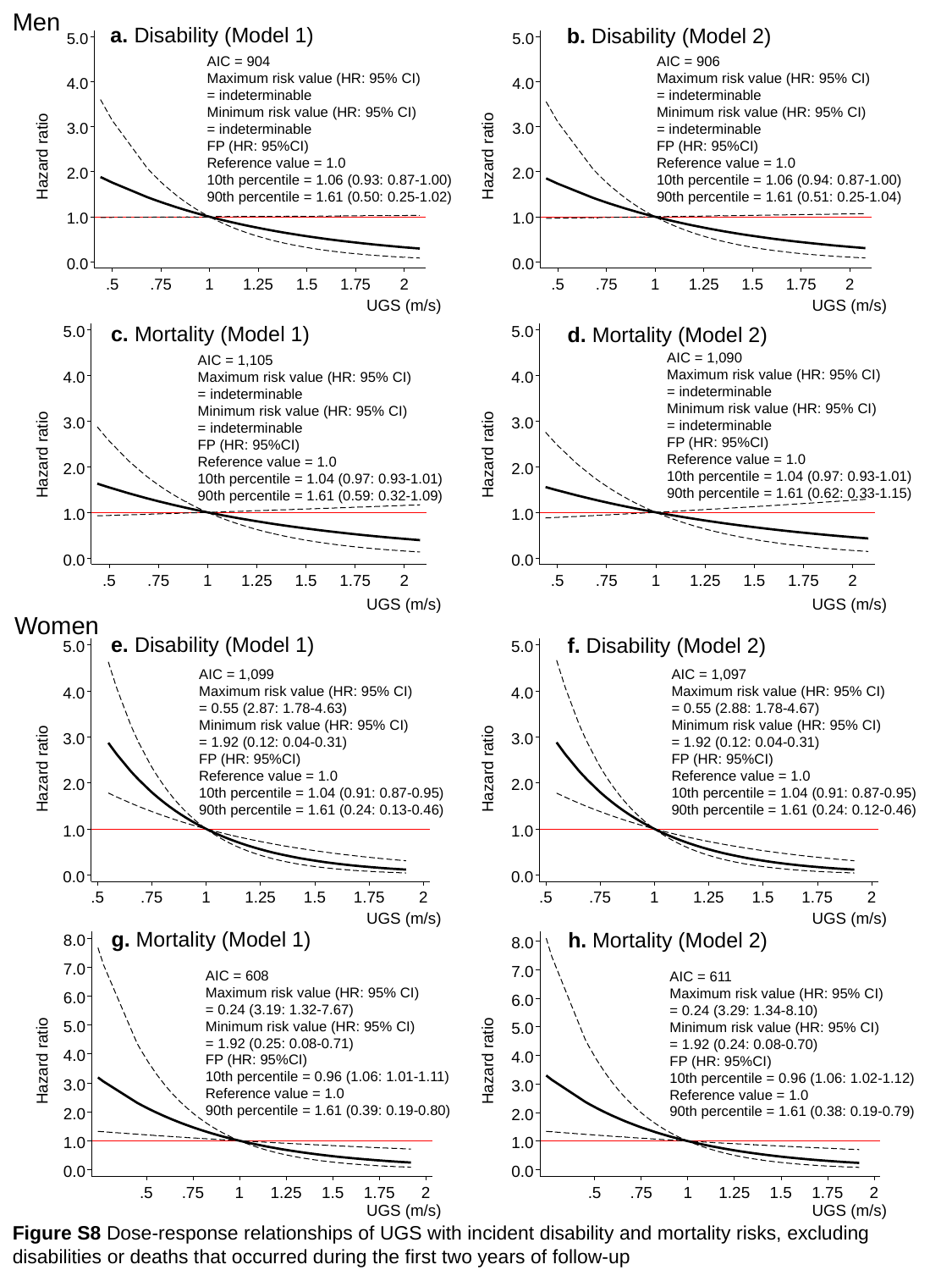

Men
a. Disability (Model 1)
b. Disability (Model 2)
AIC = 906
Maximum risk value (HR: 95% CI)
= indeterminable
Minimum risk value (HR: 95% CI)
= indeterminable
FP (HR: 95%CI)
Reference value = 1.0
10th percentile = 1.06 (0.94: 0.87-1.00)
90th percentile = 1.61 (0.51: 0.25-1.04)
AIC = 904
Maximum risk value (HR: 95% CI)
= indeterminable
Minimum risk value (HR: 95% CI)
= indeterminable
FP (HR: 95%CI)
Reference value = 1.0
10th percentile = 1.06 (0.93: 0.87-1.00)
90th percentile = 1.61 (0.50: 0.25-1.02)
Hazard ratio
Hazard ratio
UGS (m/s)
UGS (m/s)
c. Mortality (Model 1)
d. Mortality (Model 2)
AIC = 1,090
Maximum risk value (HR: 95% CI)
= indeterminable
Minimum risk value (HR: 95% CI)
= indeterminable
FP (HR: 95%CI)
Reference value = 1.0
10th percentile = 1.04 (0.97: 0.93-1.01)
90th percentile = 1.61 (0.62: 0.33-1.15)
AIC = 1,105
Maximum risk value (HR: 95% CI)
= indeterminable
Minimum risk value (HR: 95% CI)
= indeterminable
FP (HR: 95%CI)
Reference value = 1.0
10th percentile = 1.04 (0.97: 0.93-1.01)
90th percentile = 1.61 (0.59: 0.32-1.09)
Hazard ratio
Hazard ratio
UGS (m/s)
UGS (m/s)
Women
e. Disability (Model 1)
f. Disability (Model 2)
AIC = 1,099
Maximum risk value (HR: 95% CI)
= 0.55 (2.87: 1.78-4.63)
Minimum risk value (HR: 95% CI)
= 1.92 (0.12: 0.04-0.31)
FP (HR: 95%CI)
Reference value = 1.0
10th percentile = 1.04 (0.91: 0.87-0.95)
90th percentile = 1.61 (0.24: 0.13-0.46)
AIC = 1,097
Maximum risk value (HR: 95% CI)
= 0.55 (2.88: 1.78-4.67)
Minimum risk value (HR: 95% CI)
= 1.92 (0.12: 0.04-0.31)
FP (HR: 95%CI)
Reference value = 1.0
10th percentile = 1.04 (0.91: 0.87-0.95)
90th percentile = 1.61 (0.24: 0.12-0.46)
Hazard ratio
Hazard ratio
UGS (m/s)
UGS (m/s)
g. Mortality (Model 1)
h. Mortality (Model 2)
AIC = 608
Maximum risk value (HR: 95% CI)
= 0.24 (3.19: 1.32-7.67)
Minimum risk value (HR: 95% CI)
= 1.92 (0.25: 0.08-0.71)
FP (HR: 95%CI)
10th percentile = 0.96 (1.06: 1.01-1.11)
Reference value = 1.0
90th percentile = 1.61 (0.39: 0.19-0.80)
AIC = 611
Maximum risk value (HR: 95% CI)
= 0.24 (3.29: 1.34-8.10)
Minimum risk value (HR: 95% CI)
= 1.92 (0.24: 0.08-0.70)
FP (HR: 95%CI)
10th percentile = 0.96 (1.06: 1.02-1.12)
Reference value = 1.0
90th percentile = 1.61 (0.38: 0.19-0.79)
Hazard ratio
Hazard ratio
UGS (m/s)
UGS (m/s)
Figure S8 Dose-response relationships of UGS with incident disability and mortality risks, excluding disabilities or deaths that occurred during the first two years of follow-up
